# A fully open-source framework for deep learning protein real-valued distances

**DOI:** 10.1038/s41598-020-70181-0

**Published:** 2020-08-07

**Authors:** Badri Adhikari

**Affiliations:** Department of Computer Science, University of Missouri-St. Louis, St. Louis, MO 63132 USA

**Keywords:** Computational biology and bioinformatics, Structural biology

## Abstract

As deep learning algorithms drive the progress in protein structure prediction, a lot remains to be studied at this merging superhighway of deep learning and protein structure prediction. Recent findings show that inter-residue distance prediction, a more granular version of the well-known contact prediction problem, is a key to predicting accurate models. However, deep learning methods that predict these distances are still in the early stages of their development. To advance these methods and develop other novel methods, a need exists for a small and representative dataset packaged for faster development and testing. In this work, we introduce protein distance net (PDNET), a framework that consists of one such representative dataset along with the scripts for training and testing deep learning methods. The framework also includes all the scripts that were used to curate the dataset, and generate the input features and distance maps. Deep learning models can also be trained and tested in a web browser using free platforms such as Google Colab. We discuss how PDNET can be used to predict contacts, distance intervals, and real-valued distances.

## Introduction

Deep learning and covariance signals obtained from sequence alignments are accelerating the progress in the field of protein structure prediction^1^. It is exciting that information culled from sequences whose structures are not solved can serve as the primary input feature to predict contacts and distances. The most successful methods, presented in the recent CASP13 experiment (http://predictioncenter.org/), exploit such sequence databases and unanimously demonstrate that the key to enhancing the current progress is accurate contact and distance map prediction^2–4^. The distance prediction methods, in particular, are a major advancement in the area of ab initio or free modeling. Much remains to be investigated at this merging superhighway of deep learning and protein structure prediction. For example, we do not know if current deep learning methods are an ideal fit for solving the distance prediction problem. In addition, many types and combinations of features are used as inputs. These include secondary structure predictions, coevolution-based signals^5^, and raw features such as the pair frequencies matrix, the covariance matrix^6^, the compressed covariance matrix^7^, and the precision matrix^8^. How to best engineer these features for deep learning algorithms also remains an open question. How useful the solitary deep learning algorithms are compared to the classical methods based on amino acid frequencies, such as the Chou-Fasman method^9^ and the Garnier-Osguthorpe-Robson (GOR) method^10^, also remains an open question. The irony behind current methods for structure prediction is that they rely on coevolution and conservation signals in multiple sequence alignments, which a protein sequence folding in a cell does not have access to. Hence, it should be possible to build accurate models without such input features. How accurately this can be done remains to be seen. An urgent need exists for a small and representative dataset packaged for fast development and investigation; thus, we created PDNET to meet this need and fill the information gap.

**Figure 1:**
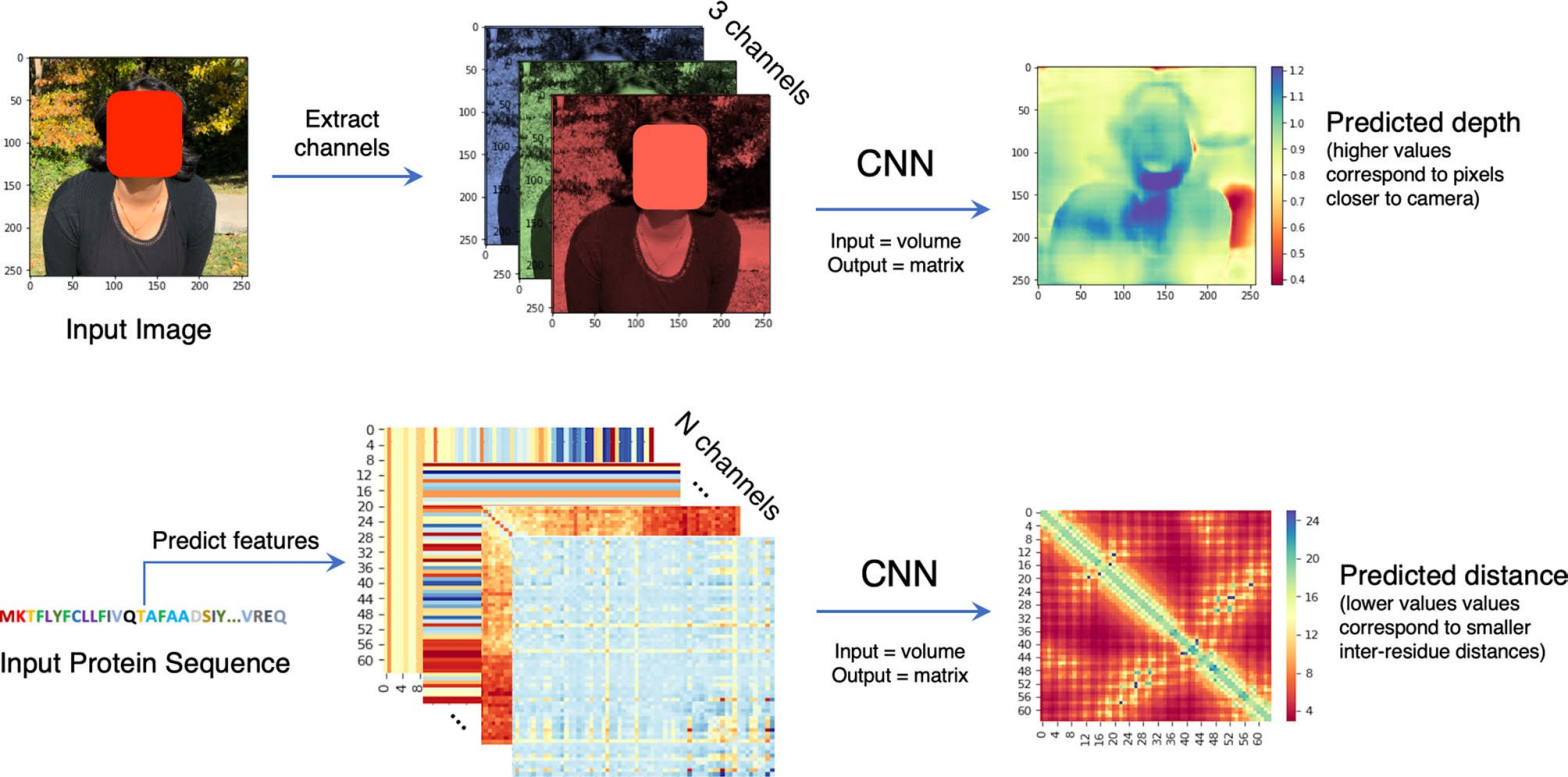
Comparison of the protein inter-residue distance prediction problem with the ‘depth prediction from a single image problem’ in computer vision. In both problems, the input to the deep learning model is a volume and the output is a 2D matrix. The depth predictions for this specific image (top right corner) were obtained with a pre-trained fully convolutional residual network (FCRN)^11^.

**Figure 2:**
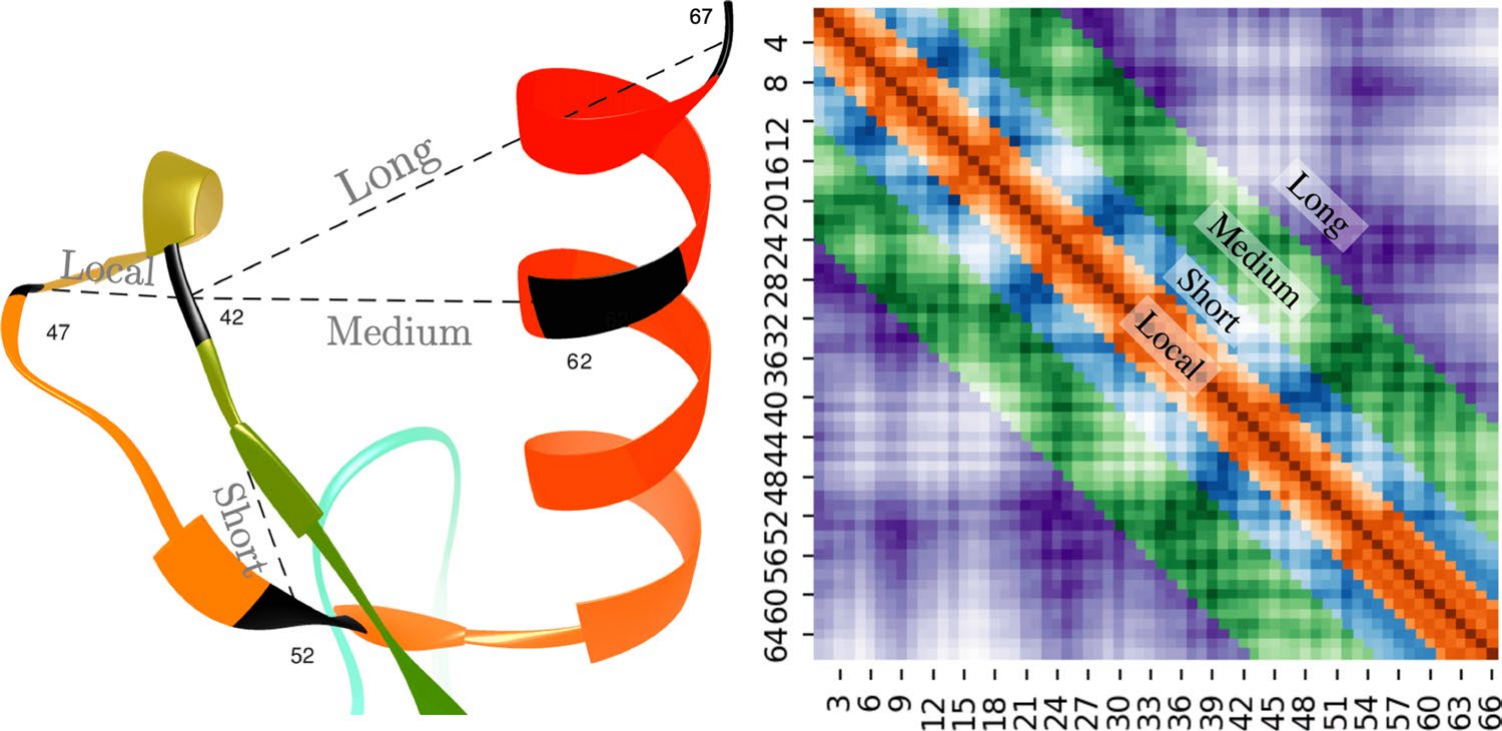
Example of long-range, medium-range, short-range, and local distances in a protein distance map. The distances between residue pairs 42–47, 42–52, 42–62, and 42–67 are examples of local, short, medium, and long-range distances, respectively (left). In the heatmap plot (right), the sequence separation domain for long, medium, and short-range distances, and local distances are [24+], [12, 23], [6, 11], and [0, 5], respectively. The labels in the x and y axis refer to the residue index in the corresponding protein sequence. The diagonal line shows that the residue pair i-i have a zero distance.

The protein inter-residue distance prediction problem is to predict a pair-wise distance matrix (2D) from a protein sequence (one-dimensional sequence of amino acids). It may be compared with the monocular or stereo depth estimation problem in computer vision as shown in Fig. 1. Image depth prediction methods such as the fully convolutional residual networks (FCRN)^11^ method take an image matrix as input and predict a depth matrix as output where each pixel has a predicted depth (distance from the camera to the object in the scene). The FCRN method is trained using various indoor and outdoor scene images. Similar to the depth prediction problem, in distance prediction, the input is a three-dimensional volume (height $\times$ width $\times$ channels) and the output is the same dimensions as the input (height $\times$ width) though with a single channel. The many characteristics of distance prediction, however, make it a unique deep learning problem. Unlike problems in computer vision, which usually have one to three input channels, the distance prediction problem may have a few to a few hundred input channels depending on what and how many input features are used. Also, a distance map is symmetrical about the diagonal line, and each pixel on the map represents a distance between a pair of residues in the sequence. A single input feature, such as a precision matrix, can have more than 400 channels. Some of these input channels are tiled rows or columns obtained from one-dimensional predictions such as secondary structures and solvent accessibility. The comparison of this problem with a computer vision problem naturally raises the question of what the convolutional filters in each layer learn. Visualization of what the deep learning layers learn and how the input features translate over the layers is difficult; thus, little research has been done in this area^12^. Because we do not know what to expect in our visualizations, exploiting the techniques of explainable artificial intelligence used in computer vision is not directly useful for the distance prediction problem. Another unique feature of the distance prediction problem, compared to computer vision problems, is that a protein structure and the corresponding distance maps cannot be augmented in the same ways that images can. For instance, distance maps cannot be scaled, rotated, or flipped. This is because an object in the real world (for example, a chair) may be tiny or large but in the case of protein structures, all proteins, small or large, are comprised of fixed size structural units such as an alpha helix. In a distance map, distance pixels away from the diagonal line are more useful for reconstructing the original structure. These distances, known as long-range distances, are also harder to predict as shown in Fig. 2. In a way, this is similar to saying that in the case of depth prediction, it is more important to predict the depth of objects closer (to the camera) in the scene than the objects that are far away. An ideal distance prediction algorithm should predict exact physical distances on the entire distance map accurately. This is extremely difficult and as such, a binary version of the problem known as contact-prediction has been more widely studied.

Although almost all currently successful methods^2, 3, 13^ formulate the distance prediction problem as a multi-class classification problem, undeniably, the ultimate goal is to accurately predict real valued distances^14^. Recently, some researchers have formulated the problem as a regression problem, which has demonstrated promising results^15^.

## Methods

### Dataset preparation

Our dataset is derived from the 3456 representative protein sequences and 150 test protein chains used to train, validate, and test the widely used DeepCov method^16^ for protein contact prediction. The 3,456 protein chains used for training and validating the DeepCov method are a subset of a set of 6,729 protein chains that have no domains homologous with the protein chains in the test set. The sequence lengths of the protein chains range from 50 to 500 residues and 50 to 266 residues in the training set and test set, respectively. The original set of 6,729 protein chains have less than 25% pairwise sequence similarity, which makes this the original set representative of the full Protein Data Bank (PDB) set. We used this list of 3,456 protein chains, the list of 150 protein chains, and the corresponding alignments to prepare our dataset. For each FASTA file in the DeepCov set, we downloaded the corresponding protein chain from http://www.pdb.org and cleaned it by removing rows containing non-standard amino acids and then renumbered the residues to match the FASTA sequence. Pairwise $C\beta$ distances between residues were calculated from the true structure (3D) to obtain a distance map (2D). The protein sequence obtained from the true structure was used as input to predict various sequence features (1D) and pairwise features (2D), which are all translated to an input volume (2D input with various channels). This input volume is the input to a deep learning model, and the output labels are the true distances. To maintain consistency with the DeepCov method, in all our experiments we selected the first 130 chains when sorted alphabetically by PDB IDs (a random subset) in the entire DeepCov set as the validation set and the remaining 3,326 chains as the training set. The alphabetical ordering ensured a random selection because PDB IDs are automatically assigned and do not have meaning^17^. Since the 150 proteins in the test set are easier (i.e. they have much richer alignments), we further tested our methods on two hard datasets—131 CAMEO-HARD (https://www.cameo3d.org/) targets released between December 8, 2018 and June 1, 2019, and the CASP13 free-modeling targets, released in 2018. These sets are also used as test datasets by other successful methods such as trRosetta^18^ to evaluate the performance of their methods. Both of these datasets serve as a test set since they were released after the original DeepCov set (development set) was curated.

Notably, while our dataset may not be a fully balanced representation of all the protein structural folds as summarized in databases such as CATH^19^, it is a representative set of the protein sequence space. Designing and developing state-of-the-art models may require a much larger dataset. However, we assert that a framework such as PDNET can answer many fundamental questions about the applicability and limitations of deep learning when solving the distance prediction problem.

### Input features

Successful methods for contact and distance prediction use a variety of features predicted and derived from the input sequence. These include predicted secondary structures, coevolution features, solvent accessibility, position-specific scoring matrix derived features, Atchley factors, many pre-computed statistical potentials, alignment statistics such as the number of effective sequences, Shannon entropy sum, mean contact potential, normalized mutual information, covariance matrices, precision matrices, etc. Contact and distance prediction methods use a variety of combinations of these features; moreover, it is not fully understood, in general, which of them contain complementary information and which are redundant. Additionally, generating all of these features for an input protein sequence is computationally expensive, both in terms of time and resources. Based on recent research advancements^18, 20, 21^, we identified the top seven features that are complementary and most informative. They are: (1) sequence profiles, (2) secondary structure predictions and (3) solvent accessibility predictions (with both 2 and 3 using PSIPRED^22^), (4) coevolutionary signals predicted using CCMpred^5^, (5) FreeContact^23^, (6) contact potentials calculated from multiple sequence alignments, and (7) Shannon entropy of the alignment column. The secondary structure predictions by PSIPRED represent the probabilities of each residue in the input sequence being a helix, strand, or coil, i.e. predicting whether each amino acid will be a part of a helix, beta-strand or a coil in the final model. Similarly, the solvent accessibility predictions by PSIPRED are binary predictions of hydrophobicity for each residue, i.e. predicting whether each amino acid will be ‘exposed’ to water or not. Looking into a predicted multiple sequence alignment, the coevolutionary predictions by CCMpred and FreeContact capture the strength of covariance between all pairs of residue positions. These predictions themselves can be considered as contact predictions. However, deep learning algorithms can improve these predictions by learning to detect noise, correct mistakes, and identify high-confidence and missing predictions^24^. With the predicted multiple sequence alignment as input, we can also calculate the contact potential matrix and the Shannon entropy sum at each residue position using the ‘alnstats’ C program in the MetaPSICOV package^25^. The potential matrix captures the frequencies of the co-varying pair positions weighted by the value of each sequence in the input sequence alignment, and the Shannon entropy sum calculates the variability at each residue position. For our last input feature, we generated sequence profiles from the multiple sequence alignments.

Features derived directly from the multiple sequence alignments (MSAs) such as covariance or precision matrix are significantly important and have achieved expected improvements^7^. However, in this work, we intentionally skipped investigating such features because working with these features requires huge storage capacities, solid state disks, and many high-end GPUs. These resources may not be available to all those wishing to use our framework. In a separate work, we will elaborately discuss the importance of all of these features (a work in progress).

### Evaluation of predicted distance maps

A primary goal of evaluating the distances in a predicted distance map is to assess their usefulness towards building full three-dimensional models. We used two sets of metrics for evaluating predicted distances: (a) the mean absolute error (MAE) of predicted distances, and (b) the precision of the contacts derived from the predicted distances. To calculate MAE, we first kept all the true distances below a certain distance threshold from the native structure, and calculated the mean absolute difference between these true distances and corresponding predicted distances. Analogous to the definition of various types of contacts, we defined local, short-range, medium-range, and long-range distances as the distances between residue pairs with sequence separation of [0, 5], [6, 11], [11, 23], and [23+] respectively. Previous studies have shown that long-range contacts, i.e., pairs separated by at least 23 residues in the sequence, are the most informative pairs for accurate reconstruction^26, 27^. Hence, we designed our evaluation metrics focusing on long-range distances (see Fig. 2). Here, we evaluate the mean absolute error of medium and long-range, as well as long-range only distances at distance thresholds of 8 Å and 12 Å ($\hbox {MAE}_{8}$ and $\hbox {MAE}_{12}$). We are certain that other variations of MAE, such as the root mean squared deviation (RMSD) can also be relevant. When evaluating the predictions for the validation set, we observed a Pearson’s correlation coefficient of 0.9 between $\hbox {MAE}_{8}$ and $\hbox {P}_{\mathrm{NC}}$.

Ideally, to obtain contacts from predicted distances, one would simply consider distances less than 8 Å as contacts. Such a technique, however, did not favor the evaluation of predicted distances. Hence, we resorted to a technique similar to the one that is currently used for evaluating predicted contacts. We assigned contact scores such that shorter predicted distances have higher scores than longer ones. A score of 0.5 is assigned for a predicted distance of 8 Å. Precisely, if $D_{ij}$ is the predicted distance between two residues, i and j, then the corresponding score $P_{ij}$ is given by:

$$\begin{aligned} P_{ij} = {\left\{ \begin{array}{ll} \frac{4.0}{D_{ij}} &{} \textit{if } D_{ij} >= 4.0 \\ 1.0 &{} \textit{otherwise}. \end{array}\right. } \end{aligned}$$

Following the practice of evaluating predicted contacts by calculating the precision of top L/x contacts, we evaluated top L, and top NC long-range contacts ($P_{L}$ and $P_{NC}$). Here L and NC stand for the length of the protein sequence and the total number of contacts in the native structure. We calculated precision as the ratio of the number of matches and the total number of contacts considered. For a protein sequence, if the corresponding true contact map does not have any long-range contacts, we exclude the target from evaluation. Our motivation for evaluating the precision of top NC contacts is driven by two insights. In a recent work^28^, we reported that selecting or evaluating the top L/x contacts does not work well for all proteins. Secondly, in the most recent CASP competition, the accessors of the contact prediction category have discussed many reasons for considering evaluation of the top NC contacts instead of fewer contacts^27^. Although the $P_{NC}$ metric is not discussed in most contact prediction method papers, we determined that as more and more accurate contact prediction methods are developed, $P_{NC}$ will emerge as a more informative, reliable, and widely adopted metric. Table 1 summarizes our evaluation metrics.

**Table 1:** Evaluation metrics used for evaluation of predicted distances and/or contacts.

Prediction	Metric	Description
Distances	$MAE_{8}$	MAE of the predicted long-range distances with corresponding true distances shorter than 8 Å
	$MAE_{12}$	MAE of the predicted long-range distances with corresponding true distances shorter than 12 Å
Contacts	$\hbox {P}_{\mathrm{L}}$	Precision of top L long-range predicted contacts
	$\hbox {P}_{\mathrm{NC}}$	Precision of top NC long-range predicted contacts

L stands for the length of the protein sequence in the native (true) structure, and NC is the number of true contacts in the structure.

### Residual network architecture

All successful methods for contact and distance prediction use residual networks and their variants^2–4, 13, 18^. We developed deep learning methods to predict contacts (PDNET-Contact), distance intervals (PDNET-Binned), and real-valued distances (PDNET-Distance), i.e., three separate models. Our deep learning architecture for contact prediction is a standard 128 block residual neural network with dropouts added in between the convolutional layers as described in our DEEPCON method^21^. Each residual block consists of the following layers: batch normalization, ReLU activation, 2D convolution using $3 \times 3$ filters, the dropout layer with a dropout rate of 0.3, another ReLU activation, and another similar 2D convolution layer. Each network has around 9.5 million parameters. For our PDNET-Contact method, we set the last layer’s activation as ‘sigmoid’ and loss was calculated using binary cross-entropy. Similarly, for PDNET-Binned, the last layer’s activation was a ‘softmax’ layer and loss was calculated using categorical cross-entropy; meanwhile, for the PDNET-Distance, the last layer’s activation was left as ReLU. We trained a model for up to 64 epochs. During each epoch of training, we randomly cropped the input feature volumes to a 128 $\times$ 128 sub-volume. Before cropping, we also padded zeros of width 5 to all sides of the input volume similar to the AlphaFold approach^3^. During prediction, however, we built a model as wide as the input sequence, i.e., we did not crop during prediction.

### Binned distance prediction

Since short (euclidean) distances between pairs with long-range separation (in sequence index) are more critical for structure reconstruction and other uses, we binned distances so that bins were narrower for shorter distances and wider for larger distances. Specifically, we used a fixed bin width of 0.2 Å for bins below 8 Å and an increasing bin width for larger distances (by a factor consisting of adding 0.2 Å for every next bin). These thresholds for the distance bins were 4, 4.2, 4.4, $\ldots$, 8, 8.4, 9.0, 9.8, 10.8, 12, $\ldots$, 21, 23.4, 26, and 26+ Å. Our technique differed slightly from the fixed-width binning technique used in methods such as RaptorX^13^ and AlphaFold^3^ and the two-width binning used in the DMPfold method^29^. We used the standard categorical cross-entropy loss to optimize our model with a softmax output layer as the last layer.

To translate the predictions into contact prediction probabilities, we summed all the probabilities in the bins below the 8 Å threshold. Similarly, to translate the predictions into distance maps, for each residue pair, we selected the distance bin with the highest probability and calculated the mean of the distance range as the predicted distance. For instance, if the highest probability bin for a residue pair is the range [6.5, 7], then the predicted distance becomes 6.75 Å.

### Real-valued distance map prediction

Predicting continuous distance values, like many other regression problems, is challenging. Considering the distance prediction as a regression problem, in particular, has a unique domain specific characteristic, i.e., it is more important to predict shorter distances more accurately than longer distances. This is because, from the perspective of structure prediction and binding-site prediction, it is more meaningful to predict inter-residue interactions than non-interactions (i.e. closer/smaller distances are more important). Andras Fiser’s group have referred to such interactions as ‘interaction hubs’^27^. It immediately follows that the commonly used machine learning loss functions such as mean squared error or mean absolute error are unfit for this purpose because they focus on optimizing the longer distance values before the shorter ones. Recently, ‘logcosh’ loss (logarithm of hyperbolic cosine) has been found to be highly effective for many problem domains. It behaves similar to the squared loss for smaller loss values and is also similar to absolute loss otherwise, i.e., the loss is not so strongly affected by the occasional incorrect predictions. However, this loss function also does not focus on optimizing shorter distances. As a solution, we propose a novel loss function that precisely focuses on optimizing the shorter distances first. The idea is to reciprocate the true and predicted distances separately and then apply the logcosh loss to the difference between the two. This ‘reciprocal’ log cosh loss is given by:

$$\begin{aligned} Loss = log\left( cosh\left( \frac{K}{P+e} - \frac{K}{T+e}\right) \right) . \end{aligned}$$

**Figure 3:**
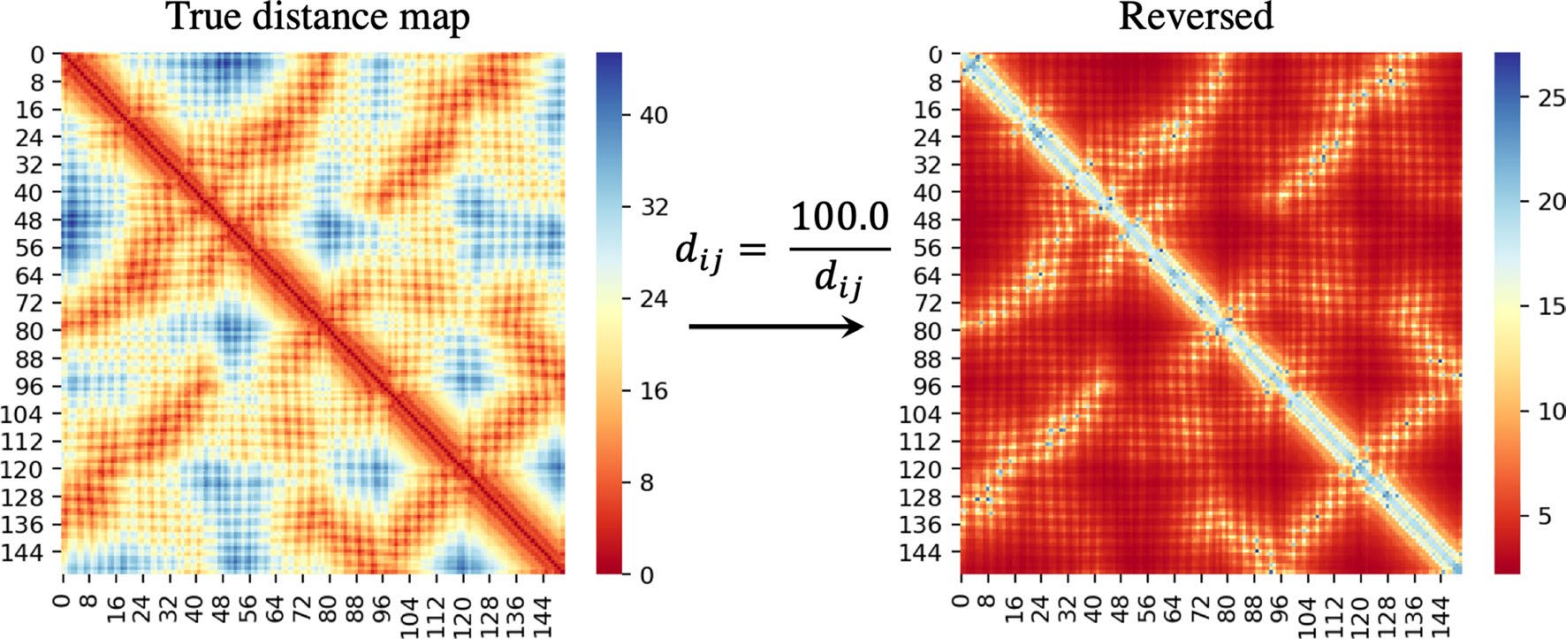
Reciprocating the distance matrix so that larger numbers represent shorter distances in the input distance matrix. The input distance matrix is shown in left and the reciprocated distance matrix is on the right. To avoid division-by-zero errors, all the diagonals in the input distance matrix are replaced by the mean of their neighbors.

Here, P is a predicted distance matrix, T is the true distance matrix, *e* is a small positive number (epsilon), and K is a scalar that scales the losses to prevent underflow. We empirically set K to 100. However, our initial implementation of this loss function in Tensorflow did not converge quickly enough. We do not fully understand why such a loss function is so efficient when implemented. As a solution, we reciprocated our distance matrices (output labels for the deep learning model) instead of reciprocating the loss function. In other words, we reciprocated the input distance matrices and used the standard logcosh loss (see Fig. 3). Such a setting made it easier for the deep learning setup to converge reliably.

## Results

### Dataset

PDNET, when zipped, is only around 1 GB in size. All the scripts used to curate the dataset, generate the input features and distance maps, as well as the scripts with deep learning architectures for training, validation and testing, are released along with the data.

### Evaluation of predicted distances and contacts

Here we present the evaluation of our deep learning methods that predict contacts (PDNET-Contact), distance intervals (PDNET-Binned), and real-valued distances (PDNET-Distance), on the 150 proteins in the test set. Overall, we determined that all methods performed relatively well on the test set because of the large and high-quality input alignments in the set, as shown in Table 2. $P_{L}$ and $P_{NC}$ for the PDNET-Contact method are 69.5% and 61.1%, respectively. The contact precision of our multi-class classification method, PDNET-Binned, is lower than our binary predictor. $P_{NC}$ for PDNET-Binned is 60.5% on the test dataset. These findings slightly contradict with the findings of Jinbo Xu’s group^30^ who found up to 4% improvement with their method based on binning. We believe that this slightly poor performance of a multi-class classification model on the mean absolute error and precision metrics is compensated by the predicted probability information for each class, which can then be used to build distogram potentials^3^ for building models. PDNET provides the platform to further improve this precision through improved architectures, hyper-parameters, and overall training techniques. For instance, we found that binning in a way such that more bins are below the 8 Å threshold can improve contact prediction precision. This is expected because, in contrast, equal-width binning implies that there will be more bins above the 8 Å threshold than below (assuming that the full range is 4–20 Å) forcing the deep learning models to equally and precisely predict the difficult-to-predict larger distance values. This, in turn, can hurt a model’s performance when the contact precision (distances only below 8 Å) is calculated.

**Table 2:** MAE and the precision of the contact prediction method (PDNET-Contact), the distance prediction method (PDNET-Distance), and binned distance prediction method (PDNET-Binned), on the 150 proteins in the test set.

Methods	$MAE_{8}$	$P_{L}$	$P_{NC}$
PDNET-Contact	–	69.5	61.1
PDNET-Distance	4.1	67.5	59.1
PDNET-Binned	3.8	67.8	60.5
DeepCov$^{{\text {a}}}$	–	55.1	–
DEEPCON	–	63.4	55.8

All MAEs are in Å, and the $P_{L}$ and $P_{NC}$ for the three PDNET methods are shown in percentage. DeepCov and DEEPCON methods’ performances, which are trained on the same dataset, are also reported.

$^{{\text {a}}}$As reported in the DeepCov^16^ paper.

**Figure 4:**
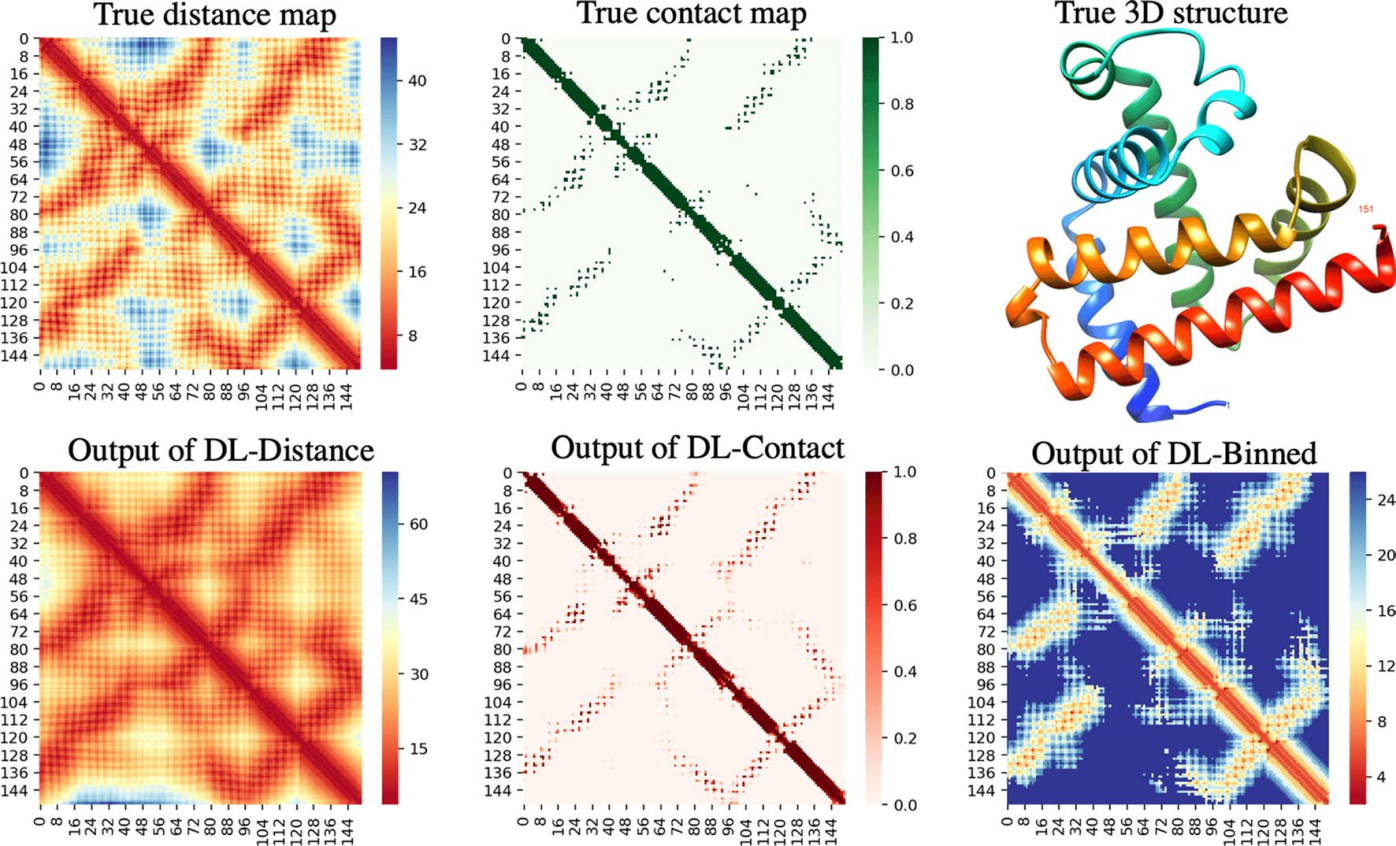
True distance map, true contact map and the native structure of ‘1a6m-A’ in the test set are shown in the top row. The output of PDNET-Distance, PDNET-Contact, and PDNET-Binned are shown in the bottom row.

Our PDNET-Distance method predicts long-range distances with an $\hbox {MAE}_{8}$ of 4.1 Å on the test set. Notably, this value seems higher because $\hbox {MAE}_{8}$ is the evaluation of all true distances below 8 Å, i.e. some incorrect predictions can significantly impact the average error. Results in Table 2 show that $P_{NC}$ of PDNET-Distance is around 3.3% (2% points) less than that of PDNET-Contact, i.e., PDNET-Distance predicts contacts with slightly less precision than PDNET-Contact. The PDNET-Distance method, however, predicts granular information contained in real-valued distance predictions which can be potentially more informative than the binary contact/non-contact prediction. As an example, in Fig. 4 we visualize and compare the predicted contacts, binned distances, and real-value distances predicted by our three methods, for the protein chain ‘1a6mA’ in the test dataset.

Next, we compared our methods with other standard methods trained on the same dataset but using covariance matrix, a slightly different input feature. Table 2 summarizes our comparison with two existing methods, DeepCov^16^ and DEEPCON^21^. These two methods only accept a covariance matrix as input. Notably, both features sets, covariance matrix or PDNET features, are derived from the same multiple sequence alignments, i.e., the input information for deriving coevolutionary signals is the same. The superior performance of all three PDNET methods compared to the DeepCov and the DEEPCON method draws attention to the fact that the seven features we selected are more predictive than the covariance matrix alone. It is obvious that adding the covariance matrix to our list of features may improve the precision. However, since covariance matrices typically have 400+ channels, the input feature files become exceptionally large (roughly 200 MB per protein chain, on average); thus, integrating the covariance matrix could make PDNET unportable. Overall, our results show that the features we have selected are more informative than the standard covariance matrix only feature.

We also trained our models to predict inter-residue $C\alpha$ (carbon-alpha) distances in addition to $C\beta$, and we did not find marked improvement in mean absolute error or contact precision. This can explain why methods such as the ones developed by Jinbo Xu’s group use separate models to predict $C\alpha -C\alpha$ or $C\gamma -C\gamma$ distances^30^. Furthermore, our PDNET-Binned method was extremely slow to train. On average, for one epoch of training with a batch size of 2, our PDNET-Binned method took around 10 hours to train, compared to our PDNET-Contact and PDNET-Distance methods, which only took 20 minutes. In general, we observed that the training time of our multi-class classifier was proportional to the number of classes or distance bins.

### Real-valued distance predictions

**Figure 5:**
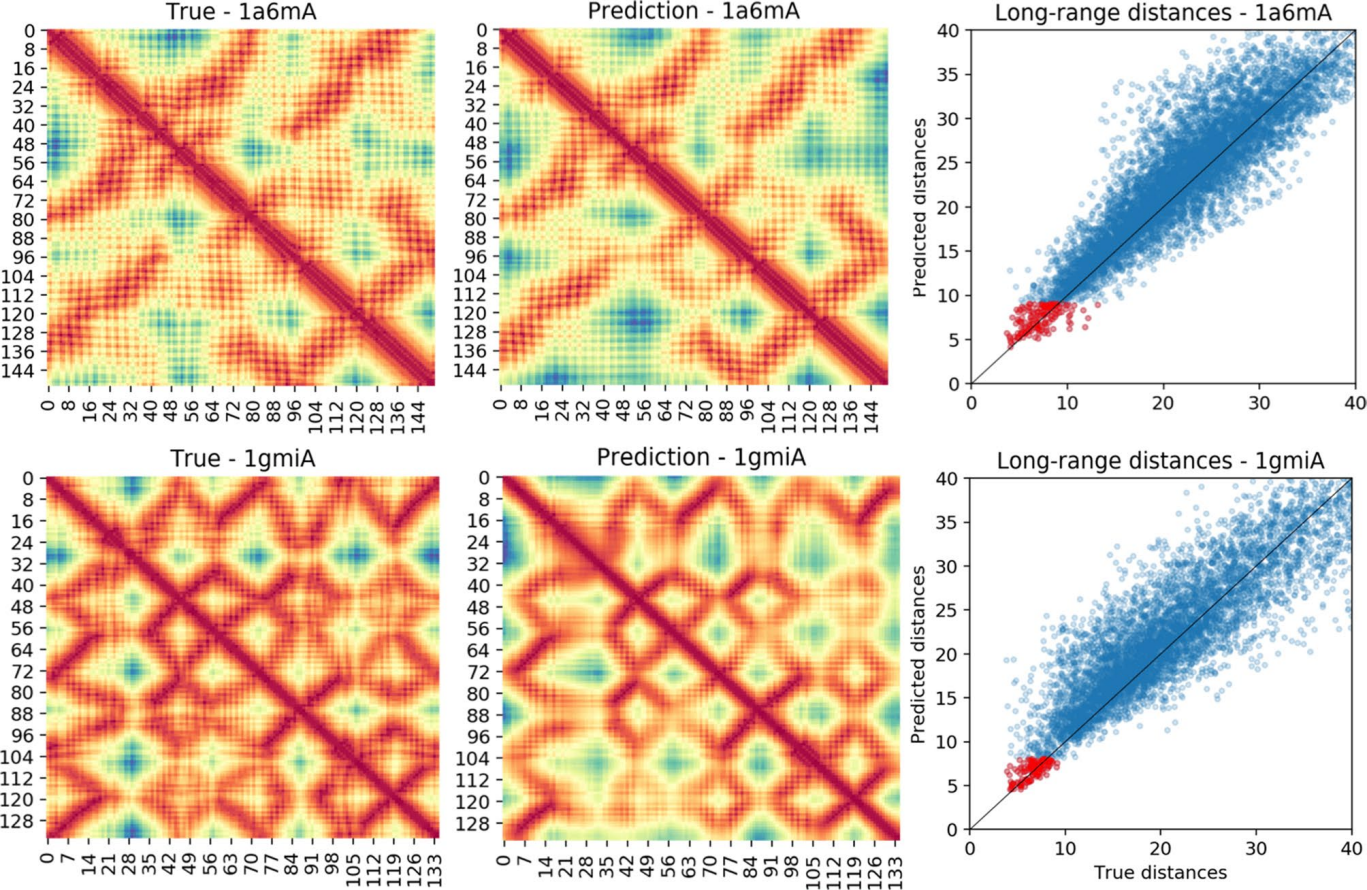
Comparison of true long-range distances and the distances predicted by our PDNET-Distance method for two random examples from the test dataset: ‘1a6m-A’ (top row) and ‘1gmi-A’ (bottom row). True distance maps and predicted distance maps are shown in the first and second columns respectively. The plots in the last column visualize the comparison of predicted long-range distances and the corresponding true distances. In these two plots, top L long-range predictions are shown in red and all other long-range distances are shown in blue.

Our real-valued distance prediction method (PDNET-Distance), trained by reciprocating the distance maps, helps the deep learning optimization focus on correctly predicting shorter distances before optimizing the longer ones. In other words, the model first attempts to predict shorter distances over others, i.e., focuses on the residue pairs that are closer in physical distance but not necessarily in sequence. To visually investigate the model’s focus on shorter distances, we randomly selected two proteins from the test set, and plotted the predicted long-range distances vs. the true long-range distances. Figure 5 shows that the model makes more precise predictions for shorter distances. These visualized examples validate that PDNET-Distance effectively focuses on correctly predicting the shortest distances over others.

### Evaluation on CASP13 and CAMEO hard targets

We further evaluated our methods on the two most difficult datasets—131 hard targets in the CAMEO competition, released after December 8, 2018, and the free-modeling targets of the CASP13 competition. Because of CASP’s agreements and policies, unlike the assessors of CASP13 and some of the top groups, we (referred to as the public) do not have access to all the native structures. Of the 32 free-modeling domains, we only had access to nine domains. Hence, we were limited in our ability to evaluate these nine domains without a way to compare with the other 23 domains. Consistent with the practice, we predicted distances and contacts for the full target and evaluated on the domains^26, 27^. For all our predictions, we used the alignments predicted by the trRosetta method, as our input. These alignment files are publicly available at https://yanglab.nankai.edu.cn/. On these nine free-modeling domains our PDNET-Contact and PDNET-Distance methods achieve $P_L$ of 38.8% and 32.3% compared to 45.0% by the RaptorX method, the top group in the competition (see Table 3). To evaluate our methods on the 131 CAMEO hard targets, we used the alignments generated using the same trRosetta method. Eight of these 131 targets’ native structure did not have any long-range contacts; thus, we skipped them and did not attempt any analysis of these targets in our evaluation. When long-range contacts were evaluated, our results showed that our PDNET-Contact and PDNET-Distance methods predicted contacts with precision similar to the trRosetta method^18^. The $P_L$ for trRosetta, PDNET-Contact, and PDNET-Distance were 48.0%, 48.3% and 46.7% respectively. Overall, this comparable performance of PDNET models with other state-of-the-art methods that use additional strategies, such as using larger training sets and model ensembling, highlight the representativeness and overall value of PDNET. It is worth noting that compared to the methods such as trRosetta, which requires many GPUs and high-speed solid state disks for training, PDNET requires lesser resources and is highly scalable. Finally, as a minor additional experiment, we cleaned the trRosetta alignments for the CAMEO targets by removing the alignment rows which are composed only of gap characters and by removing duplicate rows. We then ran our PDNET-Distance method. There were 55 such alignments out of 131. Such an alignment cleaning, we observed, did not significantly improve the contact precision, except for one protein chain, 5YVQA, for which the top L long-range precision improved from 0 to 4%.

**Table 3:** Summary of the performance of PDNET-Contact and PDNET-Distance on the 9 CASP13 free-modeling domains (for which the native structures are publicly available) and on the 131 CAMEO hard-targets.

Dataset	Methods	$P_{L}$	$P_{NC}$
CASP13 FM	PDNET-Contact	38.8	38.5
	PDNET-Distance	32.3	30.3
	Top CASP13 group	45.0	45.7
CAMEO-HARD	PDNET-Contact	48.3	45.1
	PDNET-Distance	46.7	43.7
	trRosetta	48.0	–

Performance of CASP13’s top group and the trRosetta method on the CAMEO-HARD dataset are provided as a reference. Results of the trRosetta method are copied from the Yang et al.^18^ since the predicted contacts are not publicly available.

## Discussion

Kukic et al.^14^ have previously demonstrated that reconstruction of 3D models using real-valued distances is highly accurate compared to using contacts or distance bins. Here we discuss an example using the real-valued distances predicted by PDNET-Distance and further justify why predicting real-valued distances are useful. Since a key purpose of predicted contacts and distances is to use them as restraints when building 3D models, as a random example, we picked the distance map predicted for the first protein chain (1a3aA) in the PSICOV150 test set and built two sets of 100 3D models using the Rosetta ab initio protocol^31^. The first set of models were built using contacting pairs provided as constraints (binary predictions) and the second set using the distance pairs as constraints (real-valued predictions). Comparing the best of 100 models we determined that the best model built using distance restraints was significantly more accurate with a much higher TM-score (see Supplementary Fig. 1). We translated the predicted real-valued distances to upper and lower bound constrains using an empirical rule. For a predicted real-valued distance *d*, we calculated error range, $\delta = 0.03 * d * d$, where *d* is the predicted real-valued distance, lower bound $l = d - \delta / 2$, and upper bound $u = d + \delta / 2$ (see Supplementary Fig. 2). This empirical rule of setting a larger error range for larger distances follows the design of our loss function, which focuses on learning to predict shorter (not short-range) distance values before longer ones, i.e. shorter distance predictions are more likely to be correct. We assert that the methods for calculating $\delta$ can be further optimized for a chosen 3D modeling protocol or even be predicted as an additional output channel of a deep learning model.

Next we investigated the effect of training dataset size on the precision of prediction and the importance of features used in PDNET. After selecting 11 subsets of the training set consisting of a random 100, 200, 300, 400, 500, 1,000, 1,500, 2,000, 2,500, 3,000, and 3,356 (i.e. full set) protein chains as the training dataset, we trained PDNET-Distance models and based our evaluation on the PSICOV150 test dataset. A careful analysis of the results revealed that the precision of the deep learning model starts to taper off after around 1,500 proteins in the training set (see Supplementary Fig. 3). Our results also revealed that the deep learning models trained using just a few hundred protein chains predicted remarkably precise distances. Next, to better understand the contribution from the input features used in PDNET, we grouped the seven features into five groups and reran our PDNET-Distance training by removing each feature or feature set each time. Our results showed that the CCMpred and FreeContact features are the most important input features. Removing these two features drops the top L long-range contact precision by 45% (see Supplementary Table 1). In addition to these experiments, we also tested deep learning architectures other than the DEEPCON type architecture used in the PDNET-Distance method which included: (a) a standard residual network, i.e., with no dilations and dropouts, and (b) a fully convolutional network (FCN). We observed that both residual networks perform substantially better than the FCN method, while the standard residual network performed just slightly worse than the architecture used in the PDNET-Distance method (see Supplementary Table 2).

Other groups have also attempted to ‘democratize’ the deep learning of protein distance prediction. The ProteinNet dataset by Alquraishi^32^ was released as a standardized dataset for machine learning of protein structure. It consists of large and small subsets for learning many features. Although the full dataset is four terabytes in size, smaller subsets are available for download upon request. Benchmark results for contact prediction or distance prediction are not available for this dataset. Similarly, Yun Song’s group have released TAPE^33^, a dataset packaged for predicting secondary structures, inter-residue contacts, and remote homology detection. These datasets can also be extended to predict inter-residue distances. Our work here, however, serves as the first work, to not only to discuss the distance prediction problem as the primary focus but also to release a full deep learning framework to train and evaluate distance predictions.

We believe that PDNET will be particularly helpful for researchers with some machine learning background, who are interested in difficult problems like protein structure prediction. PDNET can be easily extended to test the significance of adding other features such as the covariance matrix^16^ and the precision matrix^8^; moreover, PDNET can be used to predict dihedral angles/orientations^18^. We also believe that significant future contributions can be made by focusing on novel feature engineering techniques, loss functions, and architectures that may be particularly suitable for this specific problem of distance prediction.

## Supplementary information

Supplementary file 1

## Data Availability

All scripts, training data, deep learning code for training, validation, and testing, as well as Python Notebooks are available at https://github.com/ba-lab/pdnet/.

## References

[1] Abriata L. A, Tamò G. E, Dal Peraro M (2019). A further leap of improvement in tertiary structure prediction in casp13 prompts new routes for future assessments. Proteins: Struct. Funct. Bioinf..

[2] Kandathil S. M, Greener J. G, Jones D. T (2019). Prediction of interresidue contacts with deepmetapsicov in casp13. Proteins: Struct. Funct. Bioinf..

[3] Senior A. W (2019). Protein structure prediction using multiple deep neural networks in the 13th critical assessment of protein structure prediction (casp13). Proteins: Struct. Funct. Bioinf..

[4] Li Y, Zhang C, Bell EW, Yu D-J, Zhang Y (2019). Ensembling multiple raw coevolutionary features with deep residual neural networks for contact-map prediction in casp13. Proteins: Structure. Funct. Bioinf..

[5] Seemayer S, Gruber M, Söding J (2014). Ccmpred–fast and precise prediction of protein residue-residue contacts from correlated mutations. Bioinformatics.

[6] Kosciolek T, Jones DT (2016). Accurate contact predictions using covariation techniques and machine learning. Proteins Struct. Funct. Bioinf..

[7] Wu Q (2020). Protein contact prediction using metagenome sequence data and residual neural networks. Bioinformatics.

[8] Li Y, Hu J, Zhang C, Yu D-J, Zhang Y (2019). Respre: high-accuracy protein contact prediction by coupling precision matrix with deep residual neural networks. Bioinformatics.

[9] Chou PY, Fasman GD (1974). Prediction of protein conformation. Biochemistry.

[10] Garnier, J., Gibrat, J.-F. & Robson, B. [32] gor method for predicting protein secondary structure from amino acid sequence. In *Methods in enzymology*, vol. 266, 540–553 (Elsevier, 1996). 10.1016/s0076-6879(96)66034-0 8743705

[11] Laina, I., Rupprecht, C., Belagiannis, V., Tombari, F. & Navab, N. Deeper depth prediction with fully convolutional residual networks. In *2016 Fourth international conference on 3D vision (3DV)*, 239–248 (IEEE, 2016).

[12] Liu Y, Palmedo P, Ye Q, Berger B, Peng J (2018). Enhancing evolutionary couplings with deep convolutional neural networks. Cell Syst..

[13] Xu J (2019). Distance-based protein folding powered by deep learning. Proc. Natl. Acad. Sci..

[14] Kukic P (2014). Toward an accurate prediction of inter-residue distances in proteins using 2d recursive neural networks. BMC Bioinf..

[15] Ding, W. & Gong, H. Predicting the real-valued distances between residue pairs for proteins. arXiv preprint arXiv:1912.06306 (2019).

[16] Jones DT, Kandathil SM (2018). High precision in protein contact prediction using fully convolutional neural networks and minimal sequence features. Bioinformatics.

[17] Bank PD (1971). Protein data bank. Nat. New Biol..

[18] Yang, J. *et al.* Improved protein structure prediction using predicted interresidue orientations. *Proc. Natl. Acad. Sci.* (2020). 10.1073/pnas.1914677117 PMC6983395 31896580

[19] Dawson NL (2017). Cath: an expanded resource to predict protein function through structure and sequence. Nucleic Acids Res..

[20] AlQuraishi M (2019). End-to-end differentiable learning of protein structure. Cell Syst..

[21] Adhikari B (2020). Deepcon: protein contact prediction using dilated convolutional neural networks with dropout. Bioinformatics.

[22] McGuffin LJ, Bryson K, Jones DT (2000). The psipred protein structure prediction server. Bioinformatics.

[23] Kaján L, Hopf TA, Kalaš M, Marks DS, Rost B (2014). Freecontact: fast and free software for protein contact prediction from residue co-evolution. BMC Bioinf..

[24] Chonofsky, M., de Oliveira, S. H., Krawczyk, K. & Deane, C. M. The evolution of contact prediction: Evidence that contact selection in statistical contact prediction is changing. *BioRxiv* **660191**, (2019). 10.1093/bioinformatics/btz816 31693112

[25] Jones DT, Singh T, Kosciolek T, Tetchner S (2015). Metapsicov: combining coevolution methods for accurate prediction of contacts and long range hydrogen bonding in proteins. Bioinformatics.

[26] Schaarschmidt J, Monastyrskyy B, Kryshtafovych A, Bonvin AM (2018). Assessment of contact predictions in casp12: co-evolution and deep learning coming of age. Proteins Struct. Funct. Bioinf..

[27] Shrestha R (2019). Assessing the accuracy of contact predictions in casp13. Proteins Struct. Funct. Bioinf..

[28] Adhikari B, Cheng J (2017). Improved protein structure reconstruction using secondary structures, contacts at higher distance thresholds, and non-contacts. BMC Bioinf..

[29] Greener JG, Kandathil SM, Jones DT (2019). Deep learning extends de novo protein modelling coverage of genomes using iteratively predicted structural constraints. Nat. Commun..

[30] Xu J, Wang S (2019). Analysis of distance-based protein structure prediction by deep learning in casp13. Proteins Struct. Funct. Bioinf..

[31] Bradley P, Misura KM, Baker D (2005). Toward high-resolution de novo structure prediction for small proteins. Science.

[32] AlQuraishi M (2019). Proteinnet: a standardized data set for machine learning of protein structure. BMC Bioinf..

[33] Rao, R. *et al.* Evaluating protein transfer learning with tape. *Advances in Neural Information Processing Systems* **9686–9698**, (2019). PMC7774645 33390682

