# Supplementary material for: A fully open-source framework for deep learning protein real-valued distances
Source: Sci Rep. 2020 Aug 7;10:13374. doi: 10.1038/s41598-020-70181-0 (PMC7414848; doi:10.1038/s41598-020-70181-0)
Supplement: Supplementary file 1 [file 41598_2020_70181_MOESM1_ESM.pdf]

SUPPLEMENTARY DOCUMENT

A fully open-source framework for deep learning protein real-valued distances

Badri Adhikari
adhikarib@umsl.edu
Department of Mathematics and Computer Science
University of Missouri-St. Louis, St. Louis, MO 63132, USA

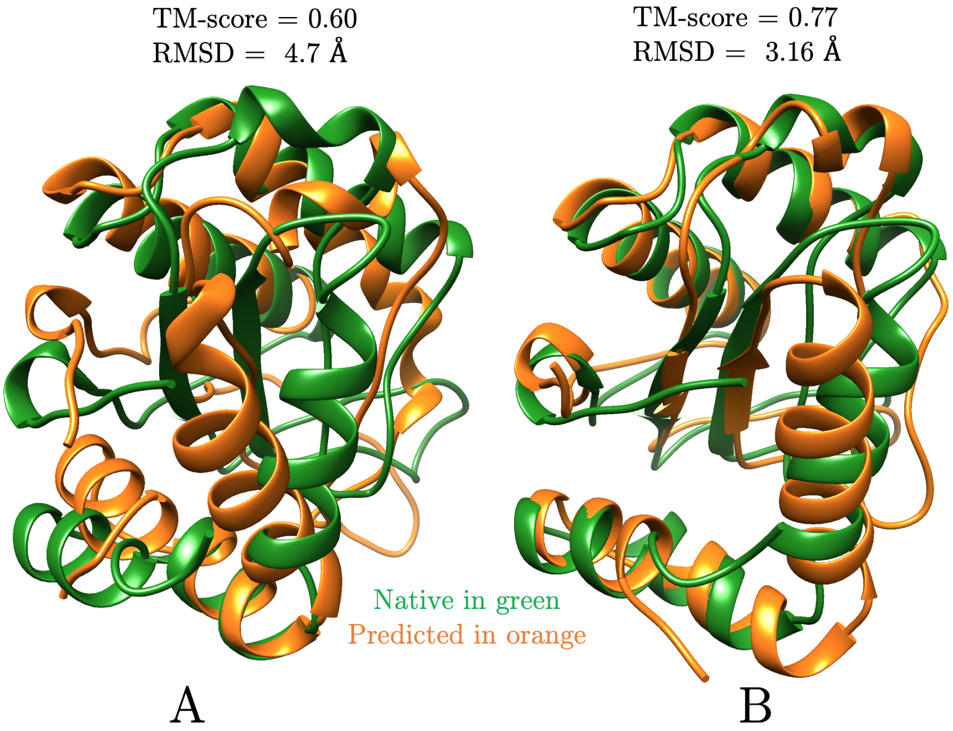

Supplementary Figure 1: Models built for the protein chain 1a3aA (first protein in the PISCOV test dataset) using contacts and distances - an example demonstrating the value of predicted real-valued distances over binary contacts. Superposition of the predicted best of 100 Rosetta model built using predicted contacts constraints (left) and distance constraints (right) with the native structure. Native structures are shown in green and predicted models in orange color. Models were visualized using the UCSF Chimera software version 1.14 available for download at https://www.cgl.ucsf.edu/chimera/.

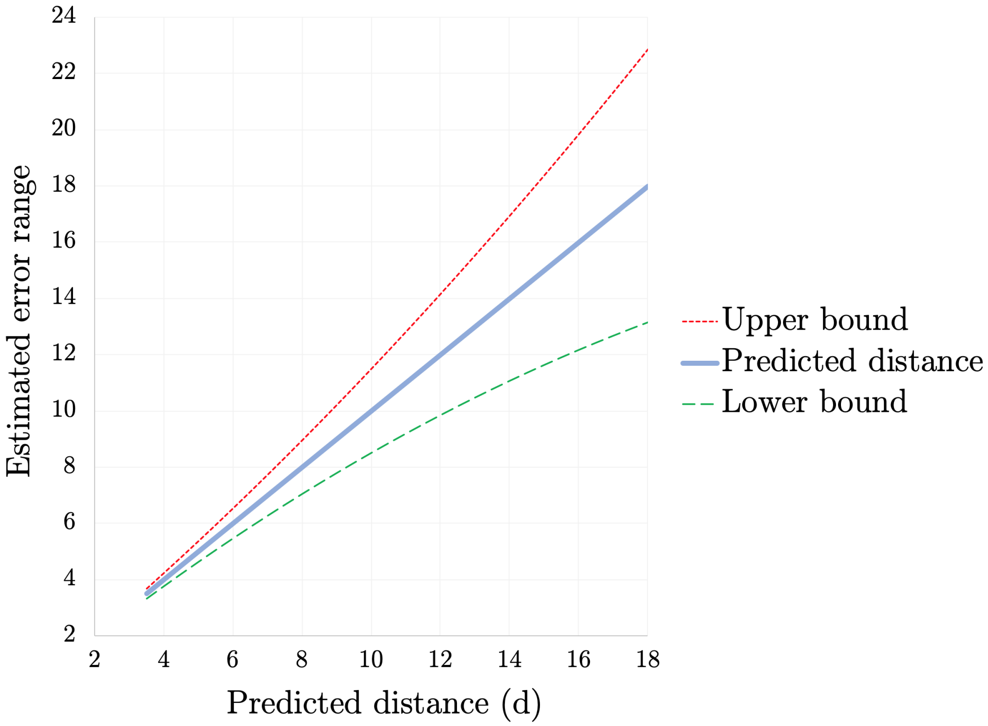

Supplementary Figure 2: Graphical representation of our empirical method to calculate upper bound (u) and lower bound (l) for a predicted distance (d) in order to generate constraints for Rosetta-based 3D modeling. All predicted distances above 18 Å are ignored while generating constraints. Here the range of bounds $\delta = 0.03 * d * d$, lower bound $l = d - \delta/2$, and upper bound $u = d + \delta/2$.

Supplementary Table 1: Study of contribution (importance) of the feature groups used in PDNET. The seven features are grouped into five with PSIPRED's secondary structure (SS) prediction and solvent accessibility (SA) predictions combined, and CCMpred and FreeContact predictions are combined. Performance evaluations using precision of top long-range contacts are reported for the PSICOV150 test set. All models are trained for 64 epochs using Callbacks to select the best model with minimum validation loss.

**Features selected for training & validation**	**Top L/5**	**Top L**	**Top Nc**
**All features (baseline)**	**92.3**	**67.5**	**59.1**
Sequence profiles removed	89.3 (-3.3%)	65.4 (-3.1%)	58.5 (-1.1%)
PSIPRED SS and SA predictions (combined) removed	91.2 (-1.2%)	66.0 (-2.1%)	58.4 (-1.3%)
CCMpred and FreeContact predictions (combined) removed	62.6 (-32.1%)	37.1 (-44.9%)	32.2 (-45.6%)
Shannon entropy of the alignment columns removed	90.7 (-1.8%)	65.1 (-3.4%)	57.1 (-3.5%)
Contact potentials from MSA removed	90.9 (-1.5%)	66.2 (-1.8%)	58.6 (-0.9%)

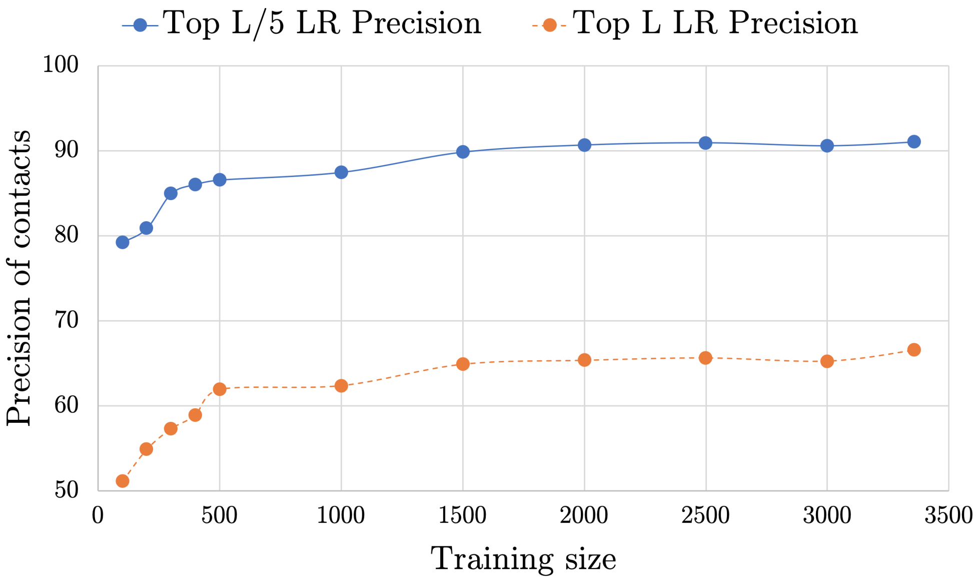

Supplementary Figure 3: Effect of training size (number of protein chains used for training) on the precision of top L/5 and top L long-range contacts on the PSICOV150 test dataset. Eleven different training jobs were run selecting random set of 100, 200, 300, 400, 500, 1000, 1500, 2000, 2500, 3000, and 3356 (all) protein chains as the training dataset. All models are trained for 64 epochs using Callbacks to select the best model with minimum validation loss.

Supplementary Table 2: Comparison of the performance of three deep learning architectures - fully convolutional network (FCN), standard residual network, and the PDNET architecture - on the PSICOV150 test dataset. Since a FCN architecture fails to learn after around 32 layers, we keep the number of convolutional layers as 32. Same number of residual blocks (128) are used to train the standard Resnet and PDNET models. All models are trained for 64 epochs using Callbacks to select the best model with minimum validation loss. Performance evaluations using precision of top long-range contacts are reported.

**Architecture**	**Top L/5**	**Top L**	**Top Nc**
FCN (Conv. Layers = 32)	87.8	59.0	51.0
Standard Resnet (Blocks = 128)	90.8	65.8	57.7
PDNET (Blocks = 128)	92.3	67.5	59.1
